# Triglyceride-glucose index is associated with symptomatic coronary artery disease in patients in secondary care

**DOI:** 10.1186/s12933-019-0893-2

**Published:** 2019-07-11

**Authors:** Alessandra da Silva, Ana Paula Silva Caldas, Helen Hermana Miranda Hermsdorff, Ângela Cristine Bersch-Ferreira, Camila Ragne Torreglosa, Bernardete Weber, Josefina Bressan

**Affiliations:** 1 Department of Nutrition and Health, Universidade Federal de Viçosa, Avenida PH Rolfs s/n, Viçosa, Minas Gerais 36570-900 Brazil; 2 Hospital for the Heart, São Paulo, SP Brazil

**Keywords:** Cardiovascular diseases, Coronary artery disease, Risk factors, Secondary care, Triglyceride-glucose index

## Abstract

**Background:**

The triglyceride-glucose index (TyG index) is a tool for insulin resistance evaluation, however, little is known about its association with coronary artery disease (CAD), which is the major cardiovascular death cause, and what factors may be associated with TyG index.

**Objective:**

To evaluate the association between the TyG index and the prevalence of CAD phases, as well as cardiovascular risk factors.

**Methods:**

The baseline data of patients in secondary care in cardiology from Brazilian Cardioprotective Nutritional Program Trial (BALANCE Program Trial) were analyzed. Anthropometric, clinical, socio-demographic and food consumption data were collected by trained professionals. The TyG index was calculated by the formula: Ln (fasting triglycerides (mg/dl) × fasting blood glucose (mg/dl)/2) and regression models were used to evaluate the associations.

**Results:**

We evaluated 2330 patients, which the majority was male (58.1%) and elderly (62.1%). The prevalence of symptomatic CAD was 1.16 times higher in patients classified in the last tertile of the TyG index (9.9 ± 0.5) compared to those in the first tertile (8.3 ± 0.3). Cardiometabolic risk factors were associated with TyG index, with the highlight for higher carbohydrate and lower lipid consumption in relation to recommendations that reduced the chance of being in the last TyG index tertile.

**Conclusion:**

The TyG index was positively associated with a higher prevalence of symptomatic CAD, with metabolic and behavioral risk factors, and could be used as a marker for atherosclerosis.

*Trial registration* ClinicalTrials.gov identifier: NCT01620398. Registered 15 June, 2012

**Electronic supplementary material:**

The online version of this article (10.1186/s12933-019-0893-2) contains supplementary material, which is available to authorized users.

## Background

Cardiovascular diseases (CVD) are a class of chronic non-communicable diseases (NCD) with the highest incidence of premature death [1] and in Brazil the CVD mortality rate is among the highest in Latin American [2]. In 2015, coronary artery disease (CAD) led to the death of 7.4 million people in the world, being one of the main causes of cardiovascular-related death [3]. The CAD occurs when the coronary arteries are blocked due to the atherosclerosis process, impairment the supply of blood to the heart muscle, which develops slowly and oftentimes, without symptoms [3, 4]. The main manifestation in its symptomatic phase is angina defined as chest pain radiating to the shoulders, arms, and jaw [5, 6].

Type 2 diabetes mellitus (T2DM) is one of the risk factors for CAD and other circulatory system diseases related to the progression and rupture of atherosclerotic plaques [7]. T2DM commonly coexist with artery hypertension and dyslipidemias, known for being endothelial aggressors [8, 9]. On the other hand, food consumption is one of the modifiable risk factors for NCD which can also influence clinical variables.

In its turn, insulin resistance (IR) contributes to the initiation of NCD, playing a key role in the development of T2DM and atherosclerosis [10]. In this sense, the triglyceride-glucose index (TyG index) has been proposed as an alternative method to evaluate IR based on only two parameters, glycemia (mg/dl) and fasting triglycerides (TG) (mg/dl) [11]. Cross-sectional studies show association between TyG index, hypertension [12], artery stiffness and coronary artery calcification [13–15]. Furthermore, contradictory results have been found in longitudinal studies. Studies have been show an association of TyG index with the incidence of CVD [16], coronary artery stenosis [17], stroke [18] and carotid atherosclerosis [19], however, no association was found for the incidence of CAD [20].

In this sense, studies that evaluate the relationship between TyG index, the prevalence of CAD and its association with CVD risk factors are scarce. Furthermore, these associations have not been studied in patients undergoing secondary care, especially among Brazilians. In view of the above, the purpose of this study was to evaluate the association between TyG index, CAD and risk factors for CVD.

## Methods

The data analyzed in this study was the baseline of the multicenter study: Brazilian Cardioprotective Nutritional Program Trial (BALANCE Program Trial) registered on ClinicalTrials.gov (NCT01620398) and coordinated by the hospital, Hospital do Coração (HCor), as part of the program “Hospitals with excellent SUS service from the “Programa de Apoio ao Desenvolvimento Institucional do Sistema Único de Saúde (PROADI-SUS)”, in partnership with the Ministry of Health of Brazil.

### Patients

Adult and elderly patients of both sexes, aged 45 years or older, who had at least one CVD in the last 10 years, were included. Patients with asymptomatic CAD were considered those that had coronary angiography or coronary angiotomography showed atherosclerotic stenosis ≥ 70% of the diameter of any coronary artery. Patients with symptomatic CAD were those with a history of angina: clinical diagnosis including diagnosis without complementary tests; the history of the positive stress test and patients with treated CAD were those with angioplasty/stent/revascularization.

All eligibility criteria are reported in the study protocol [21]. The patients signed an informed consent form before participating in the study. Note that the study was a multicenter study, therefore each center submitted its study protocol to the local Ethics Committee. The study started only after all the protocols of the centers were approved [21]. The study protocol was developed in compliance with Brazilian and international ethical principles [22].

### Data collection

Information on socioeconomic conditions (sex, age, income, level of education), behavior (food consumption, physical activity and smoking), history of diseases and medication use were obtained through structured questionnaires applied by trained interviewers. Weight, height and waist circumference (WC) (midpoint between the inferior border of the costal arch and the iliac crest in the mid axillary line) [23] were measured by trained professionals. The mean of the two WC measures (difference < 1 cm between measures) was the official waist circumference measure. Body mass index (BMI) was calculated using weight (kg)/height (m^2^). The waist–height ratio was calculated with WC (cm)/height (cm) [24]. Visceral adiposity index (VAI) was calculated by the following formulas: men = [WC (cm)/(39.69 + 1.88 × BMI (kg/m^2^))] × (TG (mmol/l)/1.03) × (1.31/HDL (mmol/l)); women = [WC (cm)/(36.58 + 1.89 × BMI (kg/m^2^))] × (TG (mmol/l)/0.81) × (1.52/HDL (mmol/l)) [25]. Blood pressure was measured one time by a trained nurse, with the mercury sphygmomanometer, following the recommendations of the American Heart Association [26].

Blood samples were collected after 12–14 h of fasting. Classical cardiovascular risk markers, such as glycemia, triglycerides (TG), total cholesterol (TC) and high-density lipoprotein (HDL-C), were measured by the enzymatic calorimetric method (Johnsons & Johnsons, Raritan, USA, VITROS 5600). Low-density lipoprotein (LDL-C) was determined by the Friedewald formula. TyG index was calculated by the formula Ln [fasting triglycerides (mg/dl) × fasting glucose (mg/dl)/2] [11].

Energy intake (kcal), carbohydrates (g), lipids (g) and protein (g) were evaluated through mean food consumption recorded by two 24-h dietary recalls. Quantitative analysis of the foods was conducted using the Nutriquanti^®^ software. The data collected were the same in all the centers participants in this study.

### Statistical analysis

The normality of the data was evaluated by the Kolmogorov–Smirnov test. The data were presented as median (interquartile range) or mean (standard deviation). Comparisons between groups were assessed by the Kruskal–Wallis test and Mann–Whitney U test. The categorical variables were evaluated by the Chi square test for linear trend. The associations between TyG index and the cardiovascular risk factors were obtained through linear regression or multinomial logistic regression (food consumption). Associations between TyG index and subtypes of CAD were obtained by Poisson regression and adjusted by possible confounders. The analysis was performed using SPSS v. 23 for Windows (SPSS, Inc., Chicago, IL, USA) and STATA^®^ 13.0. For all analyses, variations were considered statistically significant for α < 0.05.

## Results

A total of 2330 patients underwent secondary care for CVD, with a mean age of 63.2 ± 8.9 years, mean BMI of 29.1 ± 5.0 kg/m^2^ and 58.1% male.

The most prevalent CVD was treated CAD (69.0%), followed by symptomatic CAD (36.2%) and asymptomatic CAD (16.3%). Other CVD, including peripheral artery disease and stroke, corresponded respectively to 11.2 and 12.0% of total CVD.

Regarding the history of diseases, 90.3% had artery hypertension, 44.0% were diabetic and 78.2% were dyslipidemic. Furthermore, 65.1% of the included patients had a family history of CAD, 90.4%, 94.8%, 41.2% and 86.2% used anti-coagulant, antihypertensives, hypoglycemic and lipid-lowing medication, respectively.

The patients were stratified according to TyG index tertiles. Patients in the last TyG index tertile presented higher values of weight, BMI, WC, waist–height ratio, VAI, systolic blood pressure (SBP), diastolic blood pressure (DBP), TG, glycemia, total cholesterol, LDL-C, and LDL-C/HDL-C ratio compared to the first tertile of TyG index. Also, lower values of HDL-C and age were observed in those in the last tertile of TyG index. In addition, patients who were sedentary, dyslipidemic, diabetic, and hypertensive were more present in the third tertile of TyG index. Carbohydrate intake was higher in tertiles 1 and 2 compared to tertile 3, while lipid intake was higher in the third tertile than in the first tertile (Table 1).

**Table 1:** Socio-demographic, anthropometric, clinical and food consumption characteristics of patients in secondary care for cardiovascular diseases according to TyG index tertiles

Variables	TyG index tertiles			*P*-value
	1 (lowest) (n = 777)	T2 (n = 777)	3 (highest) (n = 776)	
TyG index	8.3 ± 0.3^a^	8.9 ± 0.1^b^	9.7 ± 0.5^c^	< *0.001*
Male, sex [%]	468 [34.5]	424 [31.2]	466 [34.3]	0.942
Age (years)	63.7 ± 8.8^a^	63.4 ± 8.8^a^	62.4 ± 9.2^b^	*0.008*
BMI (kg/m^2^)	27.5 ± 4.6^a^	29.4 ± 5.0^b^	30.3 ± 4.9^c^	< *0.001*
Waist circumference (cm)	95.9 ± 11.9^a^	100.2 ± 11.9^b^	103.2 ± 11.6^c^	< *0.001*
Waist-to-height ratio	0.6 ± 0.1^a^	0.6 ± 0.1^b^	0.6 ± 0.1^c^	< *0.001*
VAI	1.4 ± 0.7^a^	2.5 ± 0.9^b^	5.1 ± 4.3^c^	< *0.001*
SBP (mmHg)	129.0 ± 19.7^a^	131.8 ± 19.4^b^	131.4 ± 19.4^b^	< *0.010*
DBP (mmHg)	77.9 ± 12.5^a^	79.5 ± 12.4^b^	80.4 ± 12.6^b^	< *0.001*
Total cholesterol (mg/dl)	155.1 ± 39.0^a^	170.3 ± 43.3^b^	185.3 ± 49.4^c^	< *0.001*
HDL-C (mg/dl)	47.2 ± 14.3^a^	43.0 ± 11.3^b^	39.9 ± 9.8^c^	< *0.001*
LDL-C (mg/dl)	89.3 ± 33.8^a^	98.6 ± 38.8^b^	97.5 ± 42.5^b^	< *0.001*
LDL-C/HDL-C ratio	2.0 ± 0.9^a^	2.4 ± 0.9^b^	2.6 ± 1.2^b^	< *0.001*
Triglycerides (mg/dl)	89.9 ± 21.7^a^	145.0 ± 32.2^b^	258.2 ± 154.2^c^	< *0.001*
Glycemia (mg/dl)	95.8 ± 15.6^a^	108.0 ± 24.5^b^	151.7 ± 67.6^c^	< *0.001*
Smoking [%]	475 [33.2]	453 [31.7]	501 [35.1]	0.146
Sedentary [%]	479 [32.2]^a^	493 [33.1]^a^	516 [34.7]^b^	*0.010*
Dyslipidemic [%]	571 [31.4]^a^	589 [32.3]^a^	661 [36.3]^b^	< *0.001*
Diabetics [%]	231 [22.5]^a^	291 [28.4]^b^	504 [49.1]^c^	< *0.001*
Hypertensive [%]	676 [32.1]^a^	709 [33.7]^b^	719 [34.2]^b^	< *0.001*
Use of hypoglycemic agents [%]	205 [21.3]^a^	280 [29.1]^b^	476 [49.5]^c^	< *0.001*
Use of lipid-lowing agents [%]	660 [32.9]	679 [33.8]	669 [33.3]	0.468
Family history of CAD [%]	489 [62.9]	510 [65.8]	517 [67.1]	0.089
Calories (Kcal)	1437.5 ± 526.0	1401.2 ± 497.1	1429.3 ± 566.4	0.551
Carbohydrates (g)	189.1 ± 39.1^a^	188.5 ± 35.6^a^	183.8 ± 39.1^b^	*0.033*
Proteins (g)	70.5 ± 22.6	69.60 ± 20.6	71.5 ± 22.5	0.329
Lipids (g)	43.6 ± 12.9^a^	44.7 ± 11.9^ab^	45.7 ± 12.8^b^	*0.002*

Data are mean ± SD (standard deviation) or number [%]. P-values based in Kruskal–Wallis and Mann–Whitney U for quantitative variables and Chi square of linear trend for categorical variables

Different letters show presence of difference and equal letters show the absence of differences

Italic values show the presence of statistic significance

*DAP* diastolic blood pressure, *HDL-C* high-density lipoprotein cholesterol, *LDL-C* low-density lipoprotein, *TyG index* triglyceride-glucose index, *SBP* systolic blood pressure, *VAI* visceral adiposity index

We assessed the association between TyG index and cardiometabolic risk factors, and found that weight, BMI, WC, waist–height ratio, VAI, SBP, DBP, total cholesterol, LDL-C and LDL-C/HDL-C ratio are positively associated with TyG index, as well as the presence of dyslipidemia, diabetes, hypertension, physical inactivity and smoking. On the other hand, TyG index showed an inverse association with age and HDL-C (Additional file 1: Table S1). These results are independent of sex and medication use.

Also, food consumption was associated with TyG index. Interestingly, carbohydrate consumption above 65% of energy reduces the chance of being in the highest tertile of TyG index by 47%. On the other hand, lipid consumption below 25% of energy is associated with a 27% decrease in the chance of being in the third tertile of TyG, regardless of sex, age and use of medication (Table 2).

**Table 2:** Association between TyG index (dependent variable) with energy and macronutrient consumption in patients with heart disease

Consumption (% energy)	Model 1: TyG index tertiles			Model 2: TyG index tertiles		
	1 (lowest)	2	3 (highest)	1 (lowest)	2	3 (highest)
		OR (95% CI)			OR (95% CI)	
Energy		0.9 (0.9–1.0)	0.9 (0.9–1.0)		0.9 (0.9–1.0)	1.0 (0.9–1.0)
Carbohydrates (%)						
45–65		1 (Ref.)			1 (Ref.)	
< 45		0.9 (0.7–1.3)	1.1 (0.9–1.4)		0.9 (0.7–1.3)	1.1 (0.8–1.4)
> 65		0.7 (0.5–1.1)	*0.5* (*0.3–0.7*)		0.8 (0.5–1.1)	*0.5* (*0.3–0.8*)
Proteins (%)						
15–20	1 (Ref.)	1 (Ref.)		1 (Ref.)	1 (Ref.)	
< 15		0.9 (0.7–1.3)	0.9 (0.7–1.3)		1.0 (0.8–1.3)	1.0 (0.7–1.4)
> 20		0.9 (0.8–1.2)	1.2 (0.9–1.4)		1.0 (0.8–1.3)	1.2 (0.9–1.5)
Lipids (%)						
25–35		1 (Ref.)			1 (Ref.)	
< 25		*0.8* (*0.6–0.9*)	*0.6* (*0.5–0.8*)		0.8 (0.6–1.0)	*0.7* (*0.5–0.9*)
> 35		0.8 (0.6–1.2)	0.9 (0.7–1.2)		0.8 (0.6–1.2)	0.8 (0.6–1.1)

Data are odds ratio (95% CI) based in multinomial logistic regression

Italic values show the presence of statistic significance

Model 1: crude

Model 2: adjusted by sex, age, use of hypoglycemic, antihypertensive, anticoagulant and lipid-lowing agents

**Fig. 1:**
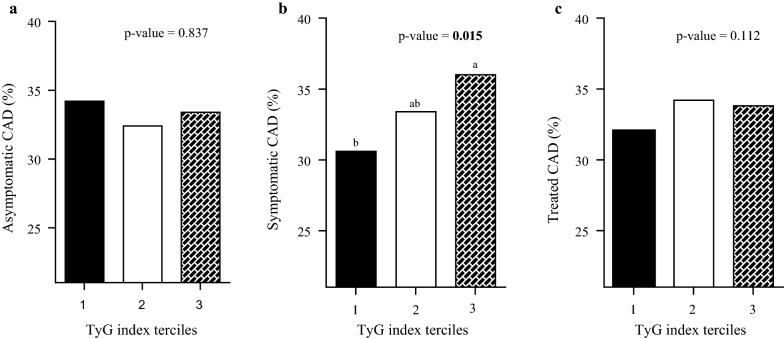
Prevalence of patients with coronary artery disease (CAD) in the phases **a** asymptomatic, **b** symptomatic and **c** treated according to TyG index tertiles

It was found that among the CVD, the prevalence of symptomatic CAD was significantly higher in the third tertile of TyG index compared to the first tertile (Fig. 1). For the other CVD (peripheral artery disease, stroke and heart attack), no differences were found among the TyG index tertiles (data not shown).

In fact, the third TyG index tertile presented a significant association with the higher prevalence of symptomatic CAD independent of the influence of social variables (sex, age), lifestyle (smoking, physical activity), clinical history (dyslipidemias, hypertension, diabetes, family history of CAD, use of medication, the respective CVD) and food consumption (calories, carbohydrates and lipids) (Table 3).

**Table 3:** Association of TyG index with the different phases of coronary artery disease

Coronary artery disease (n = 1966)	TyG index tertiles		
	1 (lowest)	2	3 (highest)
	PR (95% CI)		
Asymptomatic (n = 380)			
Model 1	Ref.	0.94 (0.75–1.18)	0.97 (0.78–1.22)
Model 2	Ref.	0.93 (0.74–1.17)	0.98 (0.79–1.23)
Model 3	Ref.	0.93 (0.74–1.17)	0.98 (0.78–1.17)
Symptomatic (n = 844)			
Model 1	Ref.	1.09 (0.95–1.25)	*1.17* (*1.03*–*1.34*)
Model 2	Ref.	1.09 (0.95–1.25)	*1.17* (*1.03–1.35*)
Model 3	Ref.	1.08 (0.94–1.24)	*1.16* (*1.01*–*1.33*)
Treated (n = 1607)			
Model 1	Ref.	1.07 (1.01–1.14)	1.05 (0.98–1.13)
Model 2	Ref.	1.07 (1.01–1.15)	1.05 (0.98–1.12)
Model 3	Ref.	1.05 (0.99–1.13)	1.03 (0.97–1.10)

Data are prevalence ratio (95% CI) based in Poisson regression

Italic values show the presence of statistic significance

Model 1: crude

Model 2: adjusted by sex and age

Model 3: adjusted by model 2, use of hypoglycemic, antihypertensive, anticoagulant, lipid-lowing agents, carbohydrate and lipids intake, stroke, peripheral artery disease, and the presence of any other stage of the disease

## Discussion

The present study investigated the relationship of TyG index with CVD and also with cardiometabolic risk factors in patients with CVD. To the best of our knowledge, this is the first study to associate TyG index with CAD in the symptomatic phase, independent of social, clinical and food consumption characteristics.

The higher prevalence of patients with symptomatic CAD in the highest TyG index tertile suggests metabolic impairment at the symptomatic stage of the disease. The association of TyG index with CAD may be related to atherosclerosis and comorbidities such as dyslipidemias, diabetes and hypertension, among others.

In clinical practice, TG and glycemia are among the classic markers of cardiometabolic risk. Alteration in the levels of these markers are directly associated with IR, progression of atherosclerosis and genesis of CVD. Despite this, few studies have explored the relationship between TyG index and CVD as well as possible influencing factors. Studies have shown that individuals in the higher TyG index quartiles were more likely to have artery stiffness [13, 27] and coronary artery calcification [15, 28] compared to those in the lower quartile. Artery stiffness and calcification of the coronary artery are processes involved in the formation and progression of atherosclerotic plaque [6], and are associated with TyG index, corroborating with our findings. Moreover, studies show an association between the highest values of TyG index, the incidence of hypertension [29, 30], T2DM [31], CVD [16, 32], subtypes of CVD such as stroke [18], as well as events due to the formation of atheroma plaque such as coronary artery stenosis [17]. Also, a study showed that the TyG index is associated with the risk of CVD development compared to the usual tool for IR evaluation [33].

In addition, we observed a positive association between TyG index and risk factors for CVD. In this sense, we showed that WC, BMI, waist–height ratio, VAI, total cholesterol, LDL-C, LDL–HDL ratio, SBP, DBP, smoking, physical inactivity, presence of hypertension and diabetes may influence TyG index. In fact, a positive association of these markers with TyG index is expected due to the compromised metabolic profile of the patients. In this context a study showed that presence of metabolic syndrome, which is characteristic of patients with metabolic impairment profile and risk factor for news diseases can be predictive of future CAD events [34]. Also, the overweight is a risk factor for CVD [35], and we showed that the BMI is associated with the TyG index, which is associated with CAD [36].

Although dietary components are considerable risk factors for CVD, to date no study has evaluated the influence of dietary intake on TyG index. Interestingly, we found that carbohydrate consumption above 65% of energy compared to current recommendation of 45 to 65% of energy, reduced the chance of being in the third tertile of TyG index, while lipid consumption < 25% of energy reduced the chance of being in the last tertile of TyG index, regardless of sex, age and medication use.

Both macronutrients when consumed in excess are associated with increased serum TG [37]; however, we found that consumption below the recommended lipid level and above the recommended carbohydrate level reduced the chances of being ranked in the highest tertile of TyG index. Current evidence suggests that in addition to quantity, the quality of lipids and carbohydrates consumed is associated with the risk of developing CVD. A meta-analysis with a high degree of evidence demonstrated that the partial substitution of saturated fatty acids with polyunsaturated fatty acids, during a minimum of 2 years, reduced the risk of cardiovascular events by 17% [38]. In addition, the substitution of saturated fats with unsaturated fats is associated with the reduced incidence of NCD [39, 40]. On the other hand, the substitution of saturated fats with carbohydrates exacerbated dyslipidemia, a risk factor for CVD [41].

We showed that carbohydrate consumption > 65% of energy reduced the chances of being in the highest TyG index tertile. A recent cohort involving 18 countries showed that carbohydrate consumption > 65% of energy was associated with a higher incidence of total mortality and mortality related to non-cardiovascular causes compared to 45–65% energy, however, the quality of carbohydrate intake was not addressed in this study [42]. The quality of carbohydrates is also associated with the development of NCD [43], such as added sugars and fructose, present mainly in industrial foods, and low fiber [44]. Studies have shown that consumption of a whole-grain diet is associated with reduced risk of CVD, cancers and total mortality [45]. In a randomized 8-week clinical trial, overweight individuals who consumed whole grains had a significant reduction in blood pressure compared to those who consumed diet based on refined grains [46]. Considering the metabolic impairment of the patients included in this study and its related risk factor, dietary intake is associated with comorbidities. Studies show that Brazilians do not consume sufficient vegetables, this trend is higher among those who present higher consumption of ultra-processed foods. Moreover, high (above recommended value) consumption of sugar, saturated fat and sodium and low (below recommended value) consumption of fiber, legumes, fruits and vegetables have been observed [47]. Carbohydrate intake above the recommended value decreases the chances of being in the third TyG index tertile, however this finding should be interpreted with caution, since the prevalence of symptomatic CAD was higher in the third tertile of TyG index compared to the first tertile.

The strength of the present study lies in it being a multicenter study which evaluated a large number of patients with CVD in all regions of Brazil. We evaluated patients with diverse eating habits despite being from the same country, and the food consumption analysis was conducted with a software which prioritized national food composition tables. However, our study has limitations. The patients were categorized as having more than one stage of CAD, however the regression model was adjusted with the presence of phases of the disease. In addition, the quality of carbohydrates, proteins and lipids were not evaluated, instead we conducted a general analysis of macronutrients.

## Conclusion

TyG index was positively associated with the higher prevalence of symptomatic CAD, regardless of social, clinical and behavioral characteristics. In addition, metabolic and behavioral risk factors are positively associated with TyG index, where carbohydrate and lipid intake, above and below recommended values respectively, decreases the chances of being at the highest TyG index tertile.

Based on the findings of this study, high TyG index is associated with symptomatic CAD, thus can serve as a complement test for the screening of patients with heart disease and an indication for therapeutic measures. Future studies should evaluate the influence of dietary components on TyG index, especially the quality of ingested macronutrients in order to elucidate the related associations.

## Additional file

**Additional file 1: Table S1.** Association between the TyG index and cardiovascular risk factors.

## Data Availability

All authors take responsibility for the integrity of the data and the accuracy of the data analysis. The datasets used and/or analyzed during the current study are available from the corresponding author on reasonable request.

## Abbreviations

CVD: cardiovascular diseases
NCD: non-communicable chronic diseases
CAD: coronary artery disease
T2DM: type 2 diabetes mellitus
IR: insulin resistance
TyG index: triglyceride-glucose index
TG: triglycerides
HCor: Hospital do Coração
PROADI-SUS: Programa de Apoio ao Desenvolvimento Institucional do Sistema Único de Saúde
WC: waist circumference
BMI: body mass index
VAI: visceral adiposity index
HDL-C: high density lipoprotein cholesterol
LDL-C: low density lipoprotein cholesterol
SBP: systolic blood pressure
DBP: diastolic blood pressure
SD: standard deviation
OD: odds ratio
PR: prevalence ratio
