# Supplementary material for: Triglyceride-glucose index is associated with symptomatic coronary artery disease in patients in secondary care
Source: Cardiovasc Diabetol. 2019 Jul 11;18:89. doi: 10.1186/s12933-019-0893-2 (PMC6625050; doi:10.1186/s12933-019-0893-2)
Supplement: Supplementary file 1 — Additional file 1: Table S1. Association between the TyG index and cardiovascular risk factors. [file 12933_2019_893_MOESM1_ESM.docx]

**Table S1:** Association between the TyG index and cardiovascular risk factors

| **Risk factors for CVD** | **β** | ***p-value*** |  | **Risk factors for CVD** | **β** | ***p-value*** |
| --- | --- | --- | --- | --- | --- | --- |
| **Waist circumference (cm)** |  |  |  | **Diastolic Blood Pressure (mmHg)** |  |  |
| Model 1 | 0.014 | **<0.001** |  | Model 1 | 0.005 | **<0.001** |
| Model 2 | 0.011 | **<0.001** |  | Model 2 | 0.004 | **<0.001** |
| **Body Mass Index (kg/m²)** |  |  |  | **LDL-C/HDL-C ratio** |  |  |
| Model 1 | 0.030 | **<0.001** |  | Model 1 | 0.133 | **<0.001** |
| Model 2 | 0.024 | **<0.001** |  | Model 2 | 0.146 | **<0.001** |
| **Waist-to-height ratio** |  |  |  | **Smoking [%]** |  |  |
| Model 1 | 2.264 | **<0.001** |  | Model 1 | 0.533 | **0.058** |
| Model 2 | 1.810 | **<0.001** |  | Model 2 | 0.062 | **0.034** |
| **Visceral Adiposity Index** |  |  |  | **Physical Inactivity [%]** |  |  |
| Model 1 | 0.153 | **<0.001** |  | Model 1 | 0.091 | **0.002** |
| Model 2 | 0.142 | **<0.001** |  | Model 2 | 0.089 | **0.003** |
| **Total cholesterol (mg/dl)** |  |  |  | **Family history of CAD [%]** |  |  |
| Model 1 | 0.004 | **<0.001** |  | Model 1 | 0.04 | 0.164 |
| Model 2 | 0.005 | **<0.001** |  | Model 2 | 0.037 | 0.185 |
| **HDL-C (mg/dl)** |  |  |  | **Dyslipidemia [%]** |  |  |
| Model 1 | -0.015 | **<0.001** |  | Model 1 | 0.207 | **<0.001** |
| Model 2 | -0.015 | **<0.001** |  | Model 2 | 0.215 | **<0.001** |
| **LDL-C (mg/dl)** |  |  |  | **Diabetes [%]** |  |  |
| Model 1 | 0.001 | **<0.001** |  | Model 1 | 0.432 | **<0.001** |
| Model 2 | 0.001 | **<0.001** |  | Model 2 | 0.434 | **<0.001** |
| **Systolic Blood Pressure (mmHg)** |  |  |  | **Hypertension [%]** |  |  |
| Model 1 | 0.001 | **<0.010** |  | Model 1 | 0.213 | **<0.001** |
| Model 2 | 0.001 | 0.136 |  | Model 2 | 0.217 | **<0.001** |

Data are β and p values based in Linear regression.

Model 1: Crude;

Model 2: Adjusted by sex, age, hypoglycemic, antihypertensive, anticoagulant and lipid-lowing agents.

HDL-C: High-density lipoprotein cholesterol; LDL-C: Low-density lipoprotein cholesterol.
